# Quantitative assessment of magnetic resonance derived perfusion measurements using advanced techniques: comparison with microspheres in an explanted pig heart system

**DOI:** 10.1186/1532-429X-15-S1-P139

**Published:** 2013-01-30

**Authors:** Andreas Schuster, Niloufar Zarinabad, Masaki Ishida, Matthew Sinclair, Jeroen P van den Wijngaard, Geraint Morton, Gilion Hautvast, Boris Bigalke, Pepijn van Horssen, Nic Smith, Jos A Spaan, Maria Siebes, Amedeo Chiribiri, Eike Nagel

**Affiliations:** 1 Division of Imaging Sciences and Biomedical Engineering, King's College London, London, UK; 2 Department of Cardiology and Pneumology and Heart Research Center, Georg-August-University, Göttingen, Germany; 3 Department of Biomedical Engineering & Physics, Academic Medical Centre, Amsterdam, Netherlands; 4 Philips Healthcare, Imaging Systems - MR, Best, Netherlands; 5 Department of Cardiology, Eberhard-Karls-University, Tübingen, Germany

## Background

Quantitative cardiovascular magnetic resonance (CMR) myocardial perfusion imaging has the potential to evolve into a routine clinical method allowing for the assessment of myocardial blood flow (MBF). Multiple quantification pathways are available based on different algorithms. These algorithms involve complex modeling and quantitative results may not necessarily be the same. At present it remains unclear which algorithm is the most accurate. An isolated perfused, magnetic resonance (MR) compatible pig heart model allows very accurate titration of MBF and in combination with high-resolution assessment of fluorescently-labeled microspheres represents a near optimal platform for validation. We sought to investigate which algorithm is most suited to quantify myocardial perfusion by CMR imaging at 1.5 and 3 Tesla using state of the art CMR perfusion techniques and quantification algorithms.

## Methods

First-pass CMR perfusion was performed in a MR compatible blood perfused pig heart model. We acquired perfusion images at resting flow (100%), 50% flow and during adenosine induced hyperemia in control and coronary occlusion conditions. MR myocardial perfusion imaging was performed at 1.5 Tesla (n=4) and at 3 Tesla (n=4). Fluorescently-labeled microspheres and externally controlled coronary blood flow served as reference standards for comparison of different quantification strategies, namely Fermi function constrained deconvolution, autoregressive moving average modeling, deconvolution using an exponential basis and deconvolution using a B-spline basis.

## Results

All CMR derived MBF estimates agreed well with microsphere results. The best correlation was achieved with Fermi function constrained deconvolution both at 1.5 Tesla (r=0.93, p<0.001) and at 3 Tesla (r=0.9, p<0.001). Fermi deconvolution correlated significantly better with the microspheres than all other methods at 3 Tesla (p<0.002, Table 2). Whilst it was superior to B-spline at 1.5 Tesla (p=0.001) it was not statistically superior to exponential deconvolution and ARMA deconvolution at 1.5 Tesla (p>0.05).The weakest correlation at 1.5 Tesla was found using B-spline deconvolution (r=0.74, p<0.001) and at 3 Tesla using exponential deconvolution (r=0.49, p<0.001).

## Conclusions

CMR derived quantitative blood flow estimates correlate with true myocardial blood flow in a controlled animal model. Amongst the different techniques, Fermi function constrained deconvolution was the most accurate at both field strengths. Quantitative CMR perfusion based on Fermi function deconvolution may therefore emerge as a useful clinical tool providing accurate blood flow assessment.

## Funding

Andreas Schuster is a British Heart Foundation (BHF) Clinical Research Fellow (FS/10/029/28253) and received grant support from the BHF (RE/08/003) and the Biomedical Research Centre (BRC-CTF 196). Matthew Sinclair receives support from the Engineering and Physical Sciences Research Council (EP/H046410/1). Jeroen P. H. M. van den Wijngaard is funded by a VENI grant of the Netherlands Organization for Scientific Research (NWO/ZonMw 916.11.171). This study was further supported by grants to the AMC from the Netherlands Heart Foundation (NHS 2006B186 and 2006B226), the Netherlands Organization for Health Research and Development (ZonMw 91105008 and 91112030), and the European Community (FP7-2007-224495: euHeart project).

Nicolas Smith receives grant support from Wellcome Trust and Engineering and Physical Sciences Research Council (EPSRC, WT 088641/Z/09/Z, EP/H046410/1, EP/G007527/2). Amedeo Chiribiri was funded by the Wellcome Trust and EPSRC under grant number WT 088641/Z/09/Z. Eike Nagel receives grant support from BHF (RE/08/003), the Wellcome Trust and Engineering and Physical Sciences Research Council (EPSRC, WT 088641/Z/09/Z) and the National Institute for Health Research (NIHR) via the comprehensive BRC award to Guy's and St Thomas' NHS Foundation Trust in partnership with King's College London and King's College Hospital NHS Foundation Trust.

